# Comparative Analysis of the Apple Root Transcriptome as Affected by Rootstock Genotype and Brassicaceae Seed Meal Soil Amendment: Implications for Plant Health

**DOI:** 10.3390/microorganisms9040763

**Published:** 2021-04-06

**Authors:** Likun Wang, Tracey S. Somera, Heidi Hargarten, Loren Honaas, Mark Mazzola

**Affiliations:** 1 Department of Plant Pathology, Washington State University, Pullman, WA 99164, USA; likun.wang@wsu.edu; 2 USDA-ARS Tree Fruit Research Lab, 1104 N. Western Ave, Wenatchee, WA 98801, USA; tracey.somera@usda.gov (T.S.S.); heidi.hargarten@usda.gov (H.H.); loren.honaas@usda.gov (L.H.); 3 Department of Plant Pathology, Stellenbosch University, Matieland 7600, South Africa

**Keywords:** apple, transcriptome, soil microbiome, Brassicaceae seed meal, replant disease

## Abstract

Brassicaceae seed meal (SM) soil amendment has been utilized as an effective strategy to control the biological complex of organisms, which includes oomycetes, fungi, and parasitic nematodes, that incites the phenomenon termed apple replant disease. Soil-borne disease control attained in response to Brassicaceae SM amendment is reliant on multiple chemical and biological attributes, including specific SM-generated modifications to the soil/rhizosphere microbiome. In this study, we conducted a comparative analyses of apple root gene expression as influenced by rootstock genotype combined with a seed meal (SM) soil amendment. Apple replant disease (ARD) susceptible (M.26) and tolerant (G.210) rootstocks cultivated in SM-amended soil exhibited differential gene expression relative to corresponding non-treated control (NTC) orchard soil. The temporal dynamics of gene expression indicated that the SM-amended soil system altered the trajectory of the root transcriptome in a genotype-specific manner. In both genotypes, the expression of genes related to plant defense and hormone signaling were altered in SM-amended soil, suggesting SM-responsive phytohormone regulation. Altered gene expression was temporally associated with changes in rhizosphere microbiome density and composition in the SM-treated soil. Gene expression analysis across the two rootstocks cultivated in the pathogen-infested NTC soil showed genotype-specific responses indicative of different defensive strategies. These results are consistent with previously described resistance mechanisms of ARD “tolerant” rootstock cultivars and also add to our understanding of the multiple mechanisms by which SM soil amendment and the resulting rhizosphere microbiome affect apple rootstock physiology. Future studies which assess transcriptomic and metagenomic data in parallel will be important for illuminating important connections between specific rhizosphere microbiota, gene-regulation, and plant health.

## 1. Introduction

Brassicaceae plant residues have been studied extensively for the capacity to control soil-borne plant pathogens and pests when applied as a soil amendment [1]. The preponderance of work has focused on the pesticidal properties of glucosinolate hydrolysis products, particularly isothiocyanates, generated in response to soil incorporation of these plant residues [2,3,4]. However, multiple complementary and/or concurrent mechanisms of action have been proposed to contribute to disease suppression in response to Brassicaceae seed meal (SM) soil amendments, including generation of ephemeral pesticidal compounds noted above, amplification of microbial elements that directly parasitize pathogens [5], increases in the abundance of microbes producing anti-microbial metabolites, and the potential induction of host defense responses [6]. For example, significant evidence exists demonstrating effective disease control of some (but not all) pathogens in response to Brassica napus SM soil amendment irrespective of production of glucosinolate hydrolysis products [7,8]. A functional microbiome of specific composition as modified by the SM amendments can be a key requirement for disease control [5,9].

Numerous studies have documented compositional changes in the soil or rhizosphere microbiome triggered by application of organic soil amendments [10,11,12,13]. Root-associated microbes can work as a whole and generate physiological changes in the host plant that result in activation of host defense responses against pathogens [14,15,16,17,18]. Similarly, long-term soil-borne disease suppression following Brassicaceae SM treatment functions, at least in part, through the resident soil microbiome [5,6,8,12]. Specific Brassicaceae seed meal formulations provided long-term suppression of the pathogen complex that incites apple replant disease (ARD), including multi-year control of the root lesion nematode, *Pratylenchus penetrans*, and *Pythium* spp. when assessed in field trials [12,19]. Disease suppression is consistently associated with amplification of specific plant beneficial components of the rhizosphere microbiome, including *Arthrobotrys* spp., *Trichoderma* spp., *Chaetomium* spp., *Humicola* spp., *Bacillus* spp., and various actinomycetes [9,12,19].

Due to the complexity of plant-soil systems, understanding of the molecular interactions between plant roots and soil-borne pathogens has lagged behind foliar pathosystems [20]. Although mechanisms contributing to disease control in response to Brassicaceae SM soil amendments have been investigated, the molecular mechanisms leading to effects on the plants themselves have not been studied extensively. Cohen et al. [6] demonstrated that apple root infection by *Rhizoctonia solani* AG-5 was suppressed when cultivated in *Brassica napus* SM-amended soil. The study indicated that the SM-mediated plant protection may be systemic and was initiated through amplification of certain components of the rhizosphere microbiome, specifically *Streptomyces* spp. While global gene expression profiling has been reported for plants in response to other soil treatments [21,22], transcriptomic studies which attempt to explore relationships between host gene expression and microbiome composition have primarily been limited to the human gut microbiome.

In addition to assessing transcriptome changes in response to planting apple in SM-amended soils, differential gene expression between the apple rootstocks G.210 and M.26 rootstocks was also evaluated in this study for plants established in non-treated control soil in order to explore genotype-dependent mechanisms affecting host tolerance. Previously, elevated transcript abundance in root tissue for many defense response genes, including genes encoding proteins involved in defense hormone signaling, was observed in the ARD tolerant apple rootstock genotype G.935 relative to the susceptible rootstock Bud.9 [23,24] when cultivated in the absence of pathogen pressure. The authors hypothesized that the tolerant genotype was better “primed” to defend against necrotrophic pathogen infection relative to the susceptible genotype. In addition, it is known that the ARD tolerant G.210 rootstock allocates a greater portion of its biomass below ground and maintains higher rates of root growth than the susceptible M.26 rootstock [25]. Therefore, resilience to root loss in G.210 may also play a pivotal role in tolerance to ARD [25,26].

In this study, it was hypothesized that: (i) genes encoding proteins that function in pathogen defense and hormone signaling are induced in response to SM application, (ii) alteration of host gene expression when cultivated in SM-treated soil is associated with changes in the rhizosphere-microbiome, and (iii) ARD-tolerance is associated with rootstock genotype-specific transcriptional changes related to more rapid defense induction, as well as modulation of root architecture.

## 2. Materials and Methods

### 2.1. Experimental Design

Soil used in this study was collected, as previously described, from a commercial orchard (GC) in Manson, WA, USA (N 47°53′05″, W 120°09′30″), possessing the diversity of elements previously shown to comprise the causal apple replant disease pathogen complex [27,28]. Seed meals utilized in this study were derived from *Brassica juncea* cv. Pacific Gold [29] and *Sinapis alba* cv. Ida Gold [30]. SMs were blended at a ratio of 1:1, passed through a 1-mm^2^ pore diameter sieve, and applied at a rate of 20 g per 2 kg of soil (= 4.4 t ha^−1^). Soil was transferred into individual 3.8-L pots after treatment and incubated on the greenhouse bench for 6 weeks to allow for degradation of potential herbicidal compounds.

Cloned M.26 and G.210 rootstock plantlets were generated by tissue culture, using previously described methods [31]. Six-week-old plantlets were planted into no treatment control (NTC) and 4.4 t ha^−1^ SM-amended soils with plants incubated under an 18/6 h day/night cycle at 24 °C. For each rootstock genotype, three individual plants were transplanted into each pot as a sample pool, with 3 replicate pots for each rootstock (M.26 and G.210) × soil treatment (control and SM 4.4 t ha^−1^) for each timepoint. Roots and rhizosphere soils were harvested at 48 h, 72 h, 7 d, and 2 months post-planting. Root samples harvested at 48 h, 72 h, and 7 d post-planting were subjected to transcriptome sequencing analysis. DNA extracted from the rhizosphere soil (described below) was used for 16S rRNA amplicon sequencing and q-PCR based microbial quantification. Plants harvested at 2 months post-planting were used for assessing plant biomass and lesion nematode root infection.

### 2.2. Treatment Effects on Plant Biomass and P. penetrans Root Density

Plant biomass was measured prior to planting and at harvest (2 months post-planting). At harvest, roots were carefully washed and a 0.5 g fine root sample was excised from each plant. Nematode extraction and quantification of *P. penetrans* root density and statistical analysis of data were conducted as previously described [19].

### 2.3. Total RNA Isolation and High-Throughput Transcriptome Sequencing

Root samples were flash frozen in liquid nitrogen and stored at −80 °C prior to RNA isolation. Tissue (2 g/sample) was ground to a fine powder in liquid nitrogen and total RNA was extracted according to method of Gasic et al. [32]. RNA samples were immediately purified using the RNeasy MinElute Cleanup Kit (Qiagen, Germantown, MD, USA) and quantified using a ND 1000 nanodrop spectrophotometer (NanoDrop Technologies, Wilmington, DE, USA). RNA integrity was assessed using Fragment Analyzer (Advanced Analytical Technologies, Ankeny, IA, USA) with the High Sensitivity RNA Analysis Kit. RNA samples with RNA Quality Numbers above 7 were used for RNA library preparation with the TruSeq Stranded mRNA Library Prep Kit (Illumina, San Diego, CA, USA). RNA sequencing was completed at the Washington State University-Spokane Genomics Core facility using Illumina sequencing (Illumina Hiseq 2500).

### 2.4. RNA-Seq Analysis

The apple reference genome Malus domestica v1.0 and associated annotation files were downloaded from Genome Database for Rosaceae (rosaceae.org, accessed on 24 February 2021 [33,34]). All Illumina transcriptome data were passed through FastQC v0.11.7 [35] per base sequence quality test, resulting in an average Phred score of 36. Data were then subjected to reference mapping and differential expression analysis using CLC Genomics Workbench (CLC GW) v9.5.3 (CLCBio, Cambridge, MA， USA). “Malus_x_domestica.v1.0.contigs.gff” was used as the reference genome and “Malus_x_domestica.v1.0.consensus_mRNA.fa” and “Malus_x_domestica.v1.0.consensus_CDS.fa” were loaded as tracks. CLC GW reference Mapper was run with high stringency, the minimum length fraction was 0.8 and the minimum similarity was 0.8. The limit for read unspecific match to Malus domestica v1.0 was set to 10. Expression values were created using the total number of reads mapped to the gene. The experiment was set up to define the relationship between samples, and the control and SM samples were compared for each rootstock genotype at each timepoint as described in Table 1.

The empirical analysis tool of differentially expressed genes (DEG) in CLC GW, which implements the “Exact Test” for two-group comparisons [36], was used to analyze DEG between control and SM treatments. Genes were described as up or down regulated in plant root tissue cultivated in SM-treated soil relative to non-treated controls. The absolute value of log2 fold change (FC) ≥ 2 and False Discovery Rate (FDR) < 0.001 were selected as stringent thresholds to minimize false positives.

The Gene Ontology (GO) and the Kyoto Encyclopedia of Genes and Genomes (KEGG) annotations were extracted from ‘Malus_x_domestica_v1.0.genes2GO” and “Malus_x_domestica_v1.0.genes2KEGG_pathways” using the match function in R (ver. 3.4.1).

Functional enrichment analysis for GO biological process terms for genes of interest was performed using Fisher’s Exact test in R with all predicted apple genes as the gene universe; alpha was set to 0.05. The annotation of protein function encoded by these genes was conducted by blastX (E-value ≤ 1.0 × e^−9^) against UniprotKB/Swiss-Prot (swissprot) database with the blast+ package ([37]; http://www.ncbi.nlm.nih.gov/BLAST/, accessed on 24 February 2021).

A separate analysis was performed to assess how G.210 and M.26 differed in their reactions when cultivated in ARD-conducive NTC soil. This analysis was performed using slightly different methodology as RNA-seq analysis procedures continue to evolve; in fact complementary methods may even be preferable [38]. Differential expression analysis of RNA-Seq data was performed on non-normalized raw counts of sequencing reads using the DESeq2 package in R version 3.5.3 [39]. All samples were of the same condition and included three biological replicates of each genotype at each timepoint. The Benjamini-Hochberg (BH) adjustment was used to calculate an adjusted *p*-value with a false discovery rate of less than 1% (P adj < 0.01). The topGO Bioconductor package in R was used to perform functional enrichment analysis on significantly upregulated and downregulated Gene Ontology (GO) terms [40]. Fisher’s exact test (algorithm = “weight01”) was used to test for statistical significance. “Malus_x_domestica_v1.0.genes2IPR.txt” was used for protein functional annotation.

### 2.5. Validation of DEG Expression Patterns by Real-Time RT-qPCR

Further analysis of 22 genes differentially expressed between NTC and SM treatments based on RNA-Seq were selected for validation with qRT-PCR. Forward and reverse primers were designed using web-based PrimerQuest Tool and oligo analyzer at Integrated DNA Technologies (idtdna.com). Primers were designed to possess an optimum annealing temperature of 60 °C, GC content of 40–60%, amplicon length of 150–200 bp, and primer length of 20–24 bp. Candidate primers were aligned to the apple genome v1.0 predicted CDS (coding sequences) using BLASTn (rosaceae.org/tools/ncbi_blast, accessed 9 April 2019) to screen for primers with off-target binding sites. 

DNase-treated RNA (2 µg) was used to synthesize first-strand cDNA using SuperScript II reverse transcriptase (Invitrogen, Grand Island, NY, USA) and poly dT (Operon, Huntsville, AL, USA) as the primer. The cDNA was diluted 20 times and a 2.4 µL aliquot was used in a 15 µL quantitative PCR (qPCR) reaction mix: 7.5 µL SYBR Green PCR Master Mix (Applied Biosystems, Warrington, UK), 0.6 µL of 10 µM forward/reverse primer (IDT, Coralville, IA, USA), and 3.9 µL nanopure water. Real-time q-PCR was conducted using a StepOnePlus Real-Time PCR System (Applied Biosystems, Foster City, CA, USA) and the following protocol: a single cycle of 10 min at 95 °C followed by 40 cycles of 15 s at 95 °C and 30 s at 55–60 °C. Relative gene expression was measured using the non-treated control group as the calibrator. No reverse transcriptase and no template negative controls were included in every PCR amplification. Each sample was represented by two independent total RNA isolations converted into two separate cDNAs. Each cDNA sample was included using three technical replicates for PCR reactions. The target gene expression was normalized to that of the internal reference gene (MdActin) using the 2^−ΔΔCT^ method [41].

### 2.6. Quantification of Microbial Density in the Rhizosphere

DNA was extracted from a 50-mg root tissue with adhering rhizosphere soil sample using the Mo-Bio plant DNA extraction kit (Mo-Bio, Carlsbad, CA, USA). Relative density of total fungi and bacteria in the samples was assessed by q-PCR. Time zero density of bacteria and fungi from plantlet roots was assessed using plantlets harvested directly from the potting mix without subsequent transplantation into soil.

The primer sets NSI1 [42]/5.8 S [43] and EU338F/EU518R [44] were used to amplify fungal ITS 1 and 5.8 S regions and bacterial 16 S rDNA region, respectively. Quantification was conducted using the StepOnePlus Real-Time PCR System, with the following protocol; initial denaturation for 10 min at 95 °C, followed by 40 cycles of 30 s at 95 °C, 30 s at 58 °C, and 30 s at 72 °C for total fungi; and denaturation for 10 min at 95 °C, followed by 40 cycles of 30 s at 95 °C and 45 s at 62 °C for total bacteria. PCR reactions contained 1 µL of 1:100 diluted DNA extract, 3 µL SYBR Green PCR Master Mix (Applied Biosystems, Warrington, UK), 0.05 µL forward/reverse primer and 5.9 µL nanopure water. Standard curves were generated for PCR quantification using purified DNA from Fusarium oxysporum isolate 1208 and Pseudomonas florescence isolate SS101 for fungi and bacteria, respectively. Dilutions were prepared to generate DNA concentrations from 0.001 to 100 pg µL-1. Each set of qPCR reactions included a no template control and all reactions were performed in triplicate.

### 2.7. 16S rRNA Sequencing of Rhizosphere DNA

PCR amplification, purification of amplicons, library preparation, and bacterial sequencing were conducted at an external facility (Molecular Research, Shallowater, TX, USA) on a MiSeq platform as previously described [19]. Final OTUs were taxonomically classified using BLASTn against a curated database derived from GreenGenes, RDPII, and NCBI (rdp.cme.msu.edu, ncbi.nlm.nih.gov, accessed 19 January 2019). Explicet software [45] was used to conduct statistical analysis and visualization of bacterial sequence data. Alpha diversity was determined using the minimum library size as the default with 1000-bootstrap resampling. Beta diversity was assessed using the Bray-Curtis similarity distance metric. A two-part test [46] was conducted after making filters for each library of soil treatment and sample time, as well as comparing them with each other with a *P* threshold of 0.05.

## 3. Results

### 3.1. Influence of Brassicaceae SM Amendment on Plant Growth and P. penetrans Root Density

**Figure 1:**
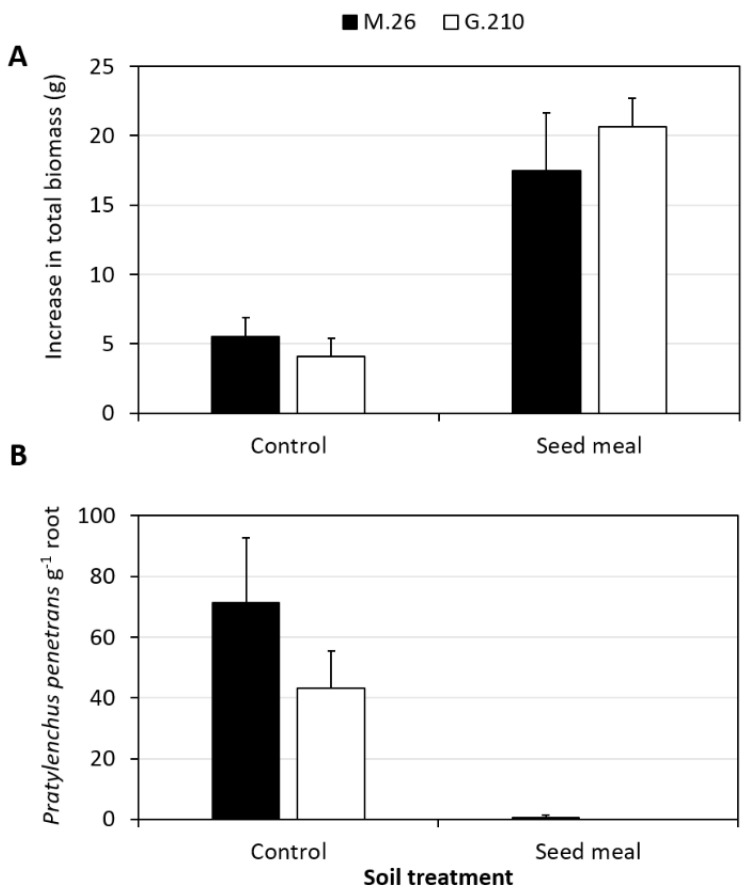
Effect of Brassicaceae seed meal soil treatment on total vegetative growth of plants (**A**) and the density of root lesion nematode, *Pratylenchus penetrans*, recovered from apple rootstock roots (**B**) at 2 months post-planting. Bars represent one standard error (*n* = 3). Seed meal = Brassicaceae seed meal formulation of *Brassica juncea/Sinapis alba* (1:1) at a rate of 4.4 t ha^−1^; Control = no treatment control.

Rootstock vegetative growth was significantly (*p* < 0.001) affected by soil treatment but not rootstock genotype (*p* = 0.736). Rootstocks grown in SM-treated soil possessed greater total biomass than rootstocks grown in NTC soil (Figure 1A). Enhanced plant biomass was associated with a significant (*p* < 0.001) reduction in *Pratylenchus penetrans* root density in SM-treated soil relative to the NTC for both rootstock genotypes (Figure 1B).

### 3.2. Mapping Statistics and Differential Gene Expression

RNA sequencing produced an average of ~33 million reads per biological replicate. Overall, 92% of the reads were mapped to the apple genome with 69% mapping to known exons (Supplementary Materials Table S1). The soil treatment effect on gene expression was compared at each timepoint for the two rootstock genotypes (Table 1). A total of 554 differentially expressed genes (DEG) were identified (FDR < 0.001, log_2_FC > 2) and differential regulation refers to SM-amendment versus the NTC. Differential gene expression overall in response to SM amendment was observed in both rootstocks, but was of greater magnitude in G.210 than in M.26, with 415 DEG and 139 DEG, respectively (Table 1). Further, the transcriptional modulation through time had a treatment-specific character. At 48 h post-planting the transcriptional response was relatively low for both genotypes (M.26 DEG = 6, G.210 DEG = 7; Table 1). For M.26, the number of DEG was greatest at 72 h (# DEG =122), yet patterns of gene expression were more convergent again by 7 d with only 10 DEG (Table 1), indicating a large amount of functional similarity between M.26 roots growing in NTC and SM-amended soils at this later timepoint. This contrasts with the transcriptional reprogramming in the G.210 rootstock that was similar in magnitude at 72 h (DEG = 177), yet continued to diverge after an additional 7 d (DEG = 232; see Table 1). Overall, the dominant signature that differentiated the rootstock treatments was a ~6-fold greater number of downregulated genes in G.210 at the end of the experiment (Table 1). 

### 3.3. Gene Ontology (GO) Enrichment Analysis of Biological Processes

#### 3.3.1. ARD Susceptible Genotype M.26

**Figure 2:**
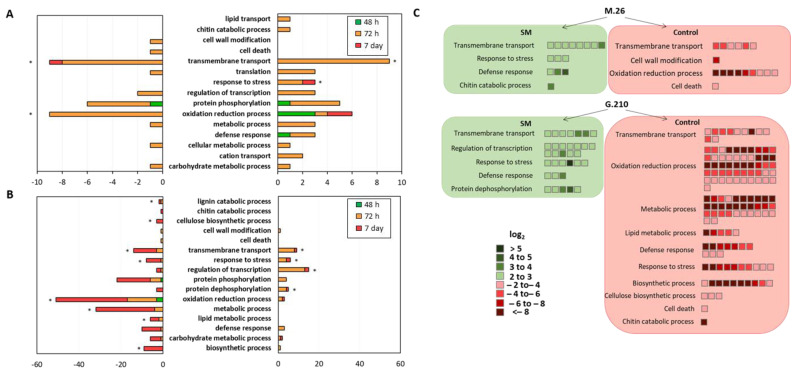
Major categories in Gene Ontology (GO) biological processes of the identified differentially expressed genes (DEG) in apple rootstocks M.26 (**A**) and G.210 (**B**); and fold changes of DEG in the major biological processes (**C**). Left panel of A and B represent processes annotated to downregulated DEG (lower in SM-amended versus control soil), and the right panel represents processes annotated to upregulated DEG (higher in SM-amended versus control soil). The X axis represents number of DEG and the Y axis indicates the selected GO biological processes. Bars with * are significantly (*p* ≤ 0.05) enriched as assessed by GO enrichment analysis.

A functional enrichment (of Gene Ontology terms) showed the GO biological process (BP) term “transmembrane transport” was significantly enriched among up- and downregulated genes in the M.26 genotype (*p* < 0.001) (Figure 2). The GO BP term “response to stress” (*p* = 0.024) was significantly enriched among upregulated genes in M.26 and the GO BP term “oxidation-reduction process” (*p* = 0.018) was enriched among downregulated genes (Figure 2A). Although “defense response” and “chitin catabolic process” were not significantly enriched, they were unique among upregulated DEG. These unique GO BP terms included genes involved in multiple aspects of plant inducible immune responses from pathogen recognition (e.g., disease resistance and pathogen recognition proteins), to signal transduction (Ca^2+^-binding proteins and protein kinases) and hormone-mediated downstream defense responses (Ethylene-responsive transcription factor) (Table 2). For example, two important serine/threonine-protein kinases, CTR1 (MDP0000198976) and PBL27 (MDP0000273596), were accumulated to higher levels in M.26 cultivated in SM treatment at 72 h post-planting (Table 2). CTR1 is a negative regulator of the ethylene (ET) response pathways in *Arabidopsis* [47], and PBL27 is involved in the signal transduction of chitin-induced immunity [48]. Among downregulated DEG in M.26, unique GO BP terms were “cell wall modification” and “cell death process” (Figure 2A). Taken together, these results provide evidence of a role for SM in modulating phytohormone signaling pathways and inducible defense responses in M.26 at 72 h post-planting. Transcriptional changes also suggest that SM-amendment was effective at reducing cellular damage in comparison to the NTC at this relatively early timepoint.

#### 3.3.2. ARD Tolerant Genotype G.210

Similar to M.26, in G.210 the GO BP terms significantly enriched in response to SM soil amendment at 72 h included “response to stress” (*p* < 0.001) and “transmembrane transport” (*p* < 0.001), but also “regulation of transcription” (*p* < 0.001), and “protein dephosphorylation” (*p* < 0.001) (Figure 2B). The GO BP Term “defense response” was also enriched among upregulated genes relative to the NTC at 72 h. Several upregulated DEG encoded proteins that may influence responses to various biotic and abiotic stressors include Abscisic acid-insensitive 5-like protein (MDP0000296303; 72 h), a CBL-interacting serine/threonine-protein kinase 9 (MDP0000216765; 72 h), and calcium sensors (Table 3).

Apple replant disease causal pathogens, *Ilyonectria, Pythium*, *Phytophthora*, *Rhizoctonia*, and *P*. *penetrans*, represent major sources of biotic stress in the rhizosphere and roots of plants. When challenged with these soil-borne pathogens, plant roots typically exhibit the JA/ET-dependent defenses [49,50,51,52], but not SA-dependent defense responses. Therefore, it is notable that two genes encoding NINJA-family proteins (MDP0000119875; MDP0000875341) which repress jasmonate responses [53], were also upregulated in G.210 at the 72-h timepoint (Table 3). Interestingly, Abscisic acid 8′-hydroxylase was 10-fold higher when G.210 was cultivated in NTC relative to SM-amended soil at 72 h. Abscisic acid (ABA) 8-hydroxylase catalyzes the first step in the oxidative degradation of (+)-ABA. In *Arabidopsis*, ABA deficiency resulted in upregulation of basal and induced transcription from JA-ethylene responsive defense genes [53]. Together, these findings point to SM-mediated suppression of JA signaling in G.210.

As mentioned above, the total number of DEG between the two soil treatments was much larger in the disease tolerant G.210 rootstock (=232) compared to the susceptible rootstock M.26 (=10) at 7 d. In contrast to the 72-h timepoint, a relatively higher number of DEG annotated with the GO BP term “defense response” were expressed in G.210 in control soils with higher disease pressure. The G.210 rootstock showed significant functional enrichment among downregulated genes for GO BP terms “oxidation reduction process” (*p* < 0.001), “response to stress” (*p* < 0.001), “transmembrane transport” (*p* = 0.036), “metabolic process” (*p* < 0.001), “biosynthetic process” (*p* = 0.001), “cellulose biosynthesis” (*p* = 0.049) and “lignin catabolic process” (*p* = 0.040; Figure 2B). Significant enrichment of the GO BP terms involved in cell wall-related processes in the NTC treatment at 7 d in G.210 (but not in M.26) may reflect genotype-specific mechanisms of resistance (i.e., high rates of root growth in G.210 = greater resilience to root loss) (Figure 2B). In addition, at 7 d, six downregulated DEG in G.210 encoded protein DMR6-like oxygenase, which functions as a negative regulatory component of SA signaling. In *Arabidopsis*, DMR6 mutants lost susceptibility to downy mildew, caused by *Hyaloperonospora arabidopsis*, and exhibited enhanced resistance to *P. syringae* and *Phytophthora capsici* [54].

### 3.4. KEGG Pathway Analysis and the Role of Secondary Metabolism

#### 3.4.1. ARD Susceptible Genotype M.26

**Figure 3:**
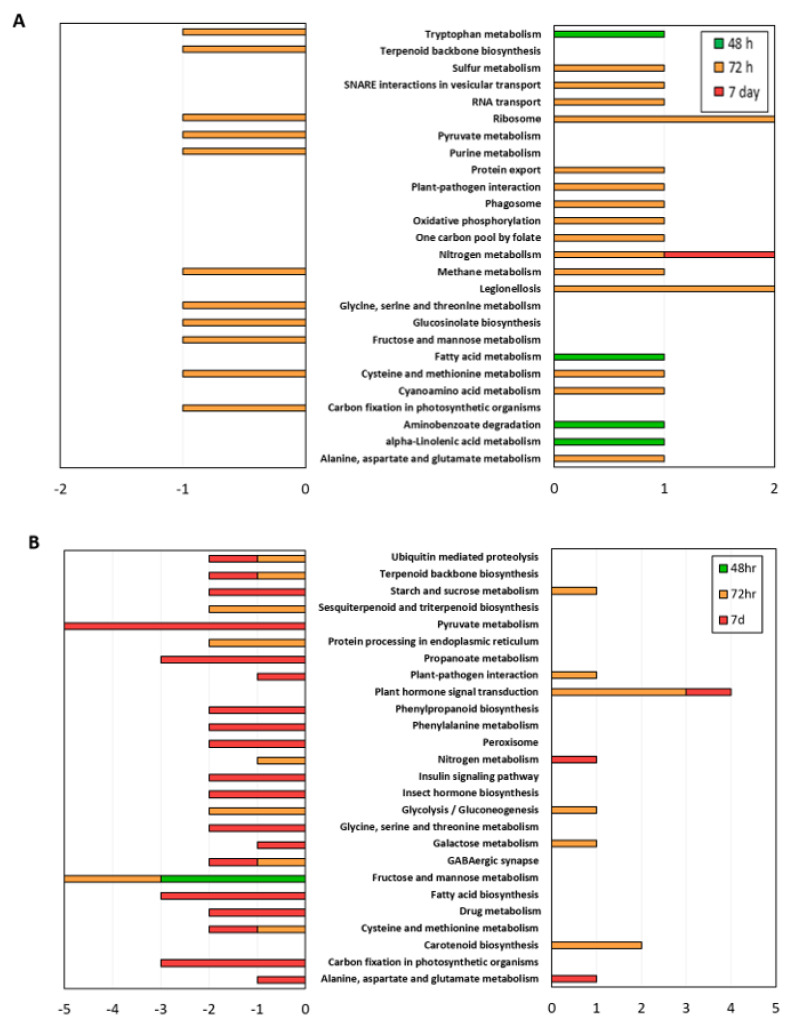
Major KEGG (Kyoto Encyclopedia of Genes and Genomes) pathways to which the identified differentially expressed genes (DEG) in apple rootstocks M.26 (**A**) and G.210 (**B**) were mapped. The left panel represents processes downregulated and right panel represents processes upregulated in the seed meal treatment. The X axis indicates number of DEG and the Y axis indicates the selected KEGG pathways.

To further understand the functions of DEG involved in metabolic and/or signal transduction pathways, all of the DEG were mapped to KEGG pathways (Figure 3). In M.26, the peak transcriptional reprogramming associated with cultivation in SM-amended soil occurred 72 h post-planting. Many upregulated DEG in M.26 mapped to KEGG pathways involved in amino acid metabolism (Figure 3A), which is tightly linked to many other metabolic pathways, including nutrient and energy metabolism, secondary metabolism, and stress response. Additionally, upregulated DEG mapped to KEGG pathways for sulfur and nitrogen metabolism. Consistent with the GO BP analysis, DEG which mapped to Plant-pathogen interaction and Phagosome were also upregulated at this time (Figure 3A). In comparison, downregulated DEG mapped to KEGG pathways for amino acid metabolism, sugar, and carbohydrate metabolism, as well as the biosynthesis of secondary metabolites (i.e., Terpenoid backbone biosynthesis). By 72 h, a number of DEG involved in phytohormone signaling and/or the utilization of secondary metabolites were upregulated in M.26 root tissue cultivated in NTC soil (Table 2). These included a putative 4-coumarate-CoA ligase (MDP0000191786) and 2 genes encoding probable S-adenosylmethionine-dependent methyltransferases (MDP0000318604 and MDP0000675952). 4-coumarate-CoA ligase contributes to JA biosynthesis, as well as to the synthesis and secretion of phenylpropanoids, while S-adenosylmethionine-dependent methyltransferase is an enzyme which methylates proteins in the biosynthesis of numerous secondary metabolites.

#### 3.4.2. ARD Tolerant Genotype G.210

In G.210, similar to M.26, upregulated DEG which mapped to defense-related KEGG pathways were identified at 72 h: Plant-pathogen interaction and Plant hormone signal transduction (Figure 3B and Table 3). Many upregulated DEG also mapped to KEGG pathways associated with carbon and energy metabolism, including starch and sucrose metabolism, glycolysis/gluconeogenesis, and galactose metabolism.

At 7 days, gene activity was significantly lower in SM treatment (86% of DEG were downregulated). Many of these downregulated DEG mapped to the same KEGG pathways to which upregulated DEG had mapped during the 72-h timepoint (e.g., starch and sucrose metabolism, nitrogen metabolism, and plant-pathogen interaction). Both DEG that mapped to Plant-pathogen interaction encoded proteins functioning in calcium ion binding. Consistent with the GO BP analysis, these findings suggest a defense response in the resistant genotype in both treatments, albeit at different times. Furthermore, the defense response in G.210 appears to have been larger in the NTC soil at 7 days than in the SM-amended soil at 72 h (Figure 3B). This is evidenced by the fold changes shown in Figure 2C, in which defense-related genes were induced to a greater degree in the roots of G.210 when grown in non-treated control soil (up to <8-fold), as compared to the SM-amended soil (up to 3–4-fold). In addition, downregulated DEG in G.210 mapped to primary metabolic pathways involved in carbohydrate and amino acid metabolism, as well as those involved in the metabolism of terpenoids, polyketides, and the biosynthesis of secondary metabolites (Figure 3B). The modulation of primary metabolic pathways to supply necessary substrates to secondary metabolic pathways is typically regulated in response to pathogen attack [55].

### 3.5. DEG Identified in Both Rootstock Genotypes Cultivated in SM Amended Soil

There were some commonalities in the gene expression response of both rootstock genotypes (DEG = 32) when cultivated in SM-amended soil. This was observed primarily after 72 h (DEG = 28) and the genes were annotated as related to disease stress response (DEG = 8) and an additional 7 DEG functional in metal ion binding, DNA binding, lipid binding, and sugar transport. Among these may be common genetic factors that play a role in SM-induced response in both genotypes (see Table 4 for details). Notably, the disease response gene MDP0000570102 is involved in flavonoid biosynthesis, which may enhance localized auxin accumulation [56,57], and DNA binding gene MDP000190029 functions in enhancing auxin biosynthesis. Flavonoids reduce the production of reactive oxygen species (ROS) and protect plants against pathogens and herbivores [58]. Thus, these unique genes may be particularly important to triggering SM-induced defense responses, as well as to the broader response of the plant to SM.

### 3.6. Validation of the Expression Patterns of Selected DEG by q-PCR

Expression patterns for 22 DEG were validated by q-PCR (Supplementary Materials Figures S1 and S2). Selected genes included those involved in various plant defense responses (MDP0000729108, MDP0000162375, MDP0000306089, MDP0000239643, MDP0000598998, MDP0000193383, MDP0000196909), cell wall modification (MDP0000281777, MDP0000231962, MDP0000411073), oxidation reduction (MDP0000629143, MDP0000214930, MDP0000258061, MDP0000181160), regulation of transcription (MDP0000296303, MDP0000581293), lipid transport (MDP0000307717), sugar transport (MDP0000212510), and protein phosphorylation (MDP0000172516). Gene expression patterns between RNA-Seq and qPCR were concordant (Supplementary Materials Figures S1 and S2).

### 3.7. Effect of SM Treatment on the Root Zone Microbial Density Post-Planting

Initially, population densities of fungi in the root zone of both rootstock cultivars were very low when cultivated in potting mix prior to transplanting in orchard soil (<1.5 pg mg^−1^ root). For both rootstocks, root zone fungal density in SM treatment did not significantly change from 0 to 72 h post-planting (<2.0 pg mg^−1^ root), but an increase was observed at 7 days. At 7 days, G.210 had roughly three times more fungal DNA per mg of root (17.1 pg mg^−1^ root) than M.26 (5.4 pg mg^−1^ root) when cultivated in SM-treated soil. The quantity of fungal DNA detected in the root zone of G.210 x NTC did not change significantly over the 7-day monitoring period (0.1–3.6 pg mg^−1^ root). As a result, after 7 days, the G.210 x SM treatment had roughly 6X more fungal DNA than the G.210 x NTC treatment. In comparison, the quantity of fungal DNA detected in the root zone of M.26 cultivated in the NTC soil increased significantly from 1.3 pg mg^−1^ root at 48 h to 22.9 pg mg^−1^ at day 7, resulting in the M.26 x SM treatment having roughly 5X less fungal DNA than the M.26 x NTC treatment. 

The quantity of bacterial DNA detected in the root zone was both time and rootstock cultivar-dependent but did not appear to be treatment-dependent. In SM-amended soil, bacterial density in the root zone was greatest in M.26 at 48 h post-transplantation (25,780 pg mg^−1^ root), with a subsequent decline observed at the 7-day timepoint (9987 pg mg^−1^ root). The same trend was observed for this rootstock when cultivated in the NTC soil with a peak density observed at 48 h (27,740 pg mg^−1^ root) and a significant decrease observed at 72 h (9543 pg mg^−1^). In contrast, bacterial density in the root zone of G.210 cultivated in SM soil exhibited a gradual increase over time from 4843 pg mg^−1^ root at time 0 (in potting soil), and a similar trend was observed in the NTC soil. For G.210 rootstock, a comparable bacterial density was detected in the root zone for both soil treatments at 7 days (NTC = 13,016 pg mg^−1^; SM = 11,052 pg mg^−1^). 

### 3.8. Effect of SM Amendment and Rootstock Genotype on Bacterial Community Composition

**Figure 4:**
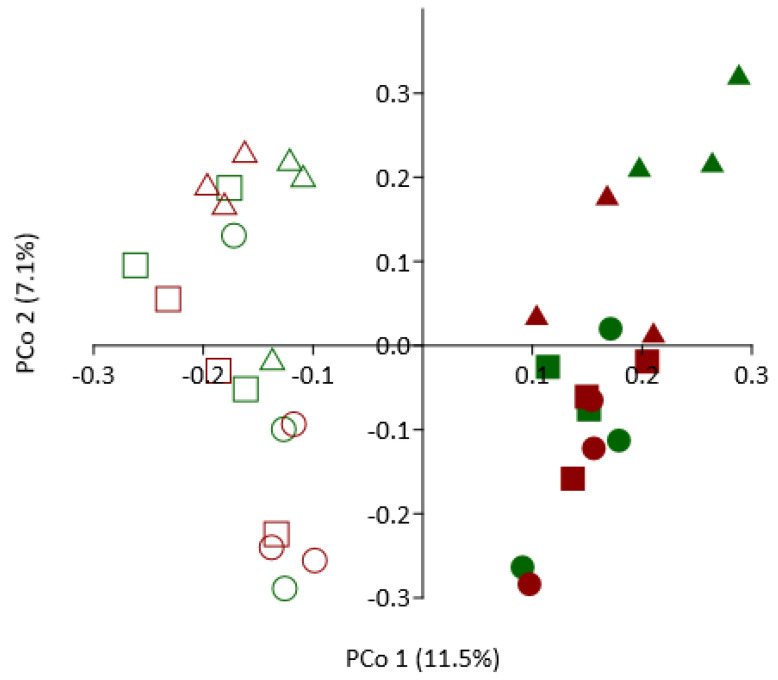
Influence of soil treatment on composition of the bacterial community detected in root/rhizosphere samples of tissue culture derived M.26 (dark red) and G.210 (green) rootstocks planted in non-treated (closed symbols) or SM-amended (open symbols) orchard replant soil. Principle coordinate analysis of operational taxonomic unit data was conducted using the Bray-Curtis dissimilarity coefficient. Symbols designate following timepoints: Circle = 48 h; square = 72 h; triangle = 7 days; across all timepoints; soil treatment seed meal versus control; *p* = 0.0001; R_ANOSIM_ = 0.6729.

**Figure 5:**
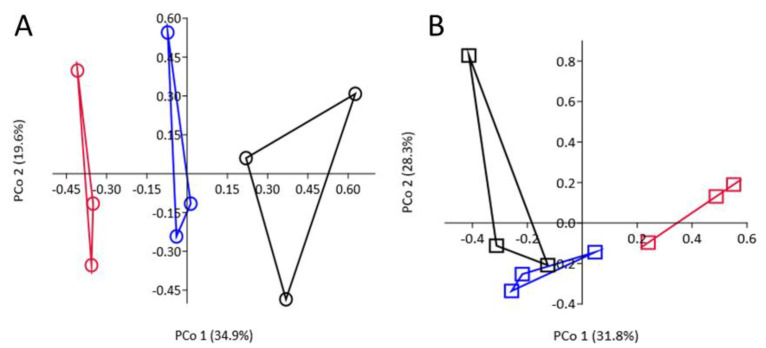
Relative changes in similarity of bacterial community composition over time in the rhizosphere off G.210 (**A**) and M.26 (**B**) apple rootstocks planted in SM (*Brassica juncea/Sinapis alba*; 1:1)-amended orchard soil. Principle coordinate analysis of operational taxonomic unit data was conducted using the Bray-Curtis dissimilarity coefficient. The convex hulls enclosing all data points for a given sampling timepoint. Black = 48 h, Blue = 72 h, and Red = 7-day timepoints.

Across all timepoints, SM treatment had a significant effect on composition of the bacterial community relative to the NTC (*p* = 0.0001 R_ANOSIM_ = 0.673; Figure 4). In the NTC soil, the root/rhizosphere bacterial community was highly similar in composition at 48-h and 72-h timepoints (R_ANOSIM_ < 0.083) and between the two rootstock genotypes (R_ANOSIM_ < −0.074). At 7 days, however, the bacterial community was highly dissimilar from that detected at the previous sampling periods, but dissimilarity was greater for G.210 (R_ANOSIM_ > 0.917) than M.26 (R_ANOSIM_ > 0.444) rootstock (Figure 4). Comparable trends were observed in the SM-amended soil for M.26 rootstock; bacterial community composition was similar at 48 h and 72 h, but, at 7 days, the community was dissimilar from that detected at the previous samplings (R_ANOSIM_ = 0.482 to 0.741; Figure 5). For G.210, root/rhizosphere bacterial communities in SM-amended soils were somewhat dissimilar between the 48-h and 72-h samples (R_ANOSIM_ = 0.371) and highly dissimilar from the community detected at 7 days (R_ANOSIM_ = 0.667 to 0.926; Figure 5).

Among bacterial phyla, Actinobacteria exhibited the largest relative increase in the root/rhizosphere of both rootstocks in response to SM treatment. At the genus level, the SM-induced increase of Actinobacteria as a proportion of the bacterial community corresponded with elevated abundance of *Streptomyces* spp., *Arthrobacter* spp., *Nocardioides* spp., and *Rhodococcus* spp. In particular, the relative proportion of *Streptomyces* spp. increased from less than 0.02% of the community to approximately 0.30% of the community in the rhizosphere of both rootstock genotypes. The number of bacterial species that were present at significantly greater abundance in the SM treatment than in the NTC increased from seven (belonging to 2 phyla) at 48 h to 37 (belonging to 5 phyla) at 7 days post-planting. The species demonstrating elevated relative abundance in SM treatment primarily belonged to the phyla Actinobacteria, Bacteroidetes, Chloroflexi, Firmicutes, Planctomycetes, and Proteobacteria, and mainly were reported to be involved in nutrient cycling or metabolizing potentially toxic organic compounds. For both rootstocks, multiple bacteria involved in nitrogen cycling, including *Devosia yakushimensis*, *Flexibacter canadensis*, *Rhizobium loessense*, *Sinorhizobium* spp., and *Devosia* spp., represented a greater proportion of the root/rhizosphere bacterial community recovered from SM-amended than the NTC soil. Bacteria known to function in suppression of various soil-borne plant pathogens, including members of the genera *Lysobacter*, *Chitinophaga*, *Bacillus*, and *Paenibacillus*, represented a significantly greater proportion of the community from both rootstocks when cultivated in SM-treated soil. *Pantoea agglomerans* comprised a significantly (*p* = 0.004) larger proportion of the bacterial community from M.26 than G.210 rootstock in the SM-treated soil.

### 3.9. Differential Gene Expression between Rootstocks in Non-Treated Control Soil

Differential gene expression between G.210 and M.26 rootstocks was investigated at each timepoint when plants were cultivated in NTC orchard replant soil. A total of 3769, 3202, and 3386 DEG were identified (*p* < 0.01) between M.26 and G.210 at 48, 72, and 7 days post-planting into NTC soil, respectively. Thus, rootstock transcriptomes were most divergent from each other at 48 h; at this time, 1823 (M.26) and 1946 (G.210) DEG were upregulated. The number of differentially expressed genes with higher expression levels was always greater in G.210 versus M.26 (48 h: 1946_G.210_, 1823_M.26_; 72 h: 1691_G.210_, 1511_M.26_; 7 days: 1909_G.210_, 1477_M.26_).

Functional analysis of over-represented GO-terms indicated that at 48 h post-planting, several biological processes related to defense were elevated in M.26 relative to G.210 (Supplementary Materials Table S2), including, wounding response, defense response to bacterium, defense response to fungi, and sphingolipid metabolic process. Other GO terms associated with genotype-specific differences in cell wall integrity which were “upregulated” in M.26 included 1-3-beta-D-glucan biosynthetic processes, catabolism of polysaccharides and L-phenylalanine, tyrosine metabolism, and lipid metabolic/biosynthetic processes. L-phenalylalanine catabolism and lipid metabolic/biosynthetic processes may also be associated with reprogramming the transcriptome towards secondary metabolite production. 

**Figure 6:**
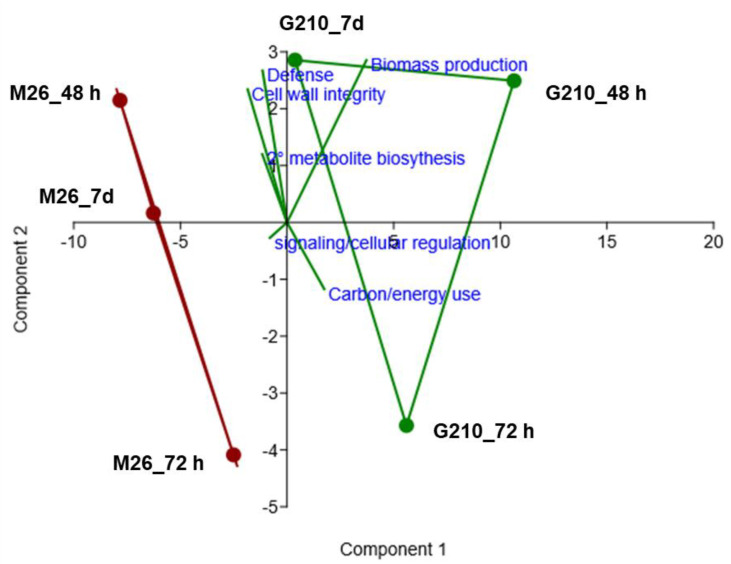
Principal components analysis of over-represented GO-terms terms in M.26 or G.210 rootstock at 48 h, 72 h, and 7 days post-planting into non-treated orchard replant soil (*p*-values derived from topGO gene enrichment analysis). For each genotype x time combination, significantly enriched GO-terms were tallied according to their predicted involvement in the following roles: defense, cell wall integrity, secondary metabolite production, signaling/cellular recognition, biomass production, and carbon/energy use. Only GO-terms with a *p*-value < 0.05 and a False Discovery Rate (FDR) of < 1% were counted. This data was then used as input for the PCA. Convex hulls are shown for each genotype; G.210 (green), M26 (dark red).

Over-represented GO terms (Supplementary Materials Tables S2 and S3) were assigned to 6 broad categories: cell wall integrity, defense, secondary metabolite biosynthesis, signaling/cellular regulation, biomass production, and carbon/energy use. PCA was then used to visualize the information contained in the enriched GO terms according to genotype and time (Figure 6). The samples show the largest amount of variation along PC1 (~70%), indicating that a greater amount of variance in the data set is explained by genotype than by time. In addition, defense, cell wall integrity, and secondary metabolite biosynthesis were highly correlated with each other and negatively correlated with biomass production, as well as carbon/energy use.

At all three timepoints, DEG potentially involved in cellular glucan metabolic processes were overrepresented in G.210 relative to M.26. The majority of DEG assigned to this GO term were involved in cell wall synthesis processes and included (1) UDP-forming Alpha-1,4-glucan-protein synthases, (2) Cellulose synthase proteins, and (3) Glycoside hydrolases family 16. Several UDP-glucose/GDP-mannose dehydrogenases were also expressed at higher levels at all timepoints in G.210. In plants, this enzyme is important for the synthesis of hemicellulose and pectin, which are the components of newly formed cell walls (InterPro v.73). This may be related to a need to synthesize biomass components for rapidly dividing root cells. Other aspects of new biomass production that were higher in G.210 relative to M.26 included genes involved in protein synthesis and DNA replication. At the same time, genes involved in protein ubiquitination (degradation) and translesion synthesis (DNA repair) were significantly upregulated in M.26 relative to G.210.

At both 48 and 72 h, there was strong evidence of differential transcriptional programming between the two genotypes in regards to carbon/energy use. The TCA cycle, gluconeogenesis, glycolysis, and acyl-CoA metabolic processes were initially “downregulated” in M.26 relative to G.210 (Figure 6, Supplementary Materials Table S3). Similarly, most of the enzymes involved in the conventional TCA cycle were identified as being downregulated in M.26 relative to G.210, including isocitrate dehydrogenase, oxoglutarate/iron-dependent oxygenase, fumarate lyase, malate dehydrogenase, and malic oxidoreductase. By 72 h post-planting, additional biological processes related to carbon/energy use partitioning became elevated in G.210 relative to M.26: NAD biosynthesis, the pentose-phosphate shunt, and glutamate and glutamine biosynthesis. During this timepoint (72 h), specific signaling-related processes (signal transduction, protein farnesylation, and protein homooligomerization) also became elevated in G.210 relative to M.26. Protein farnesylation has been shown to play a specific role in plant defense signaling [59].

As stated above, several biological processes related to defense were elevated in M.26 relative to G.210 48 h post-planting in the NTC soil. In contrast, by 7 days, many GO terms related to defense were enriched in G.210 relative to M.26, including defense response, response to biotic stimulus, chitin catabolic processes, and peroxisome fission. Figure 6 reflects these transcriptional changes, as G.210 × 7 days is positioned more closely to the top right quadrant of the plot. This result suggests that G.210 began directing resources towards additional defense mechanisms, including secondary metabolite biosynthesis, at a later time than M.26 in NTC soil.

## 4. Discussion

### 4.1. Rootstock Transcriptome Responses in SM Treated Soil

Soil-borne disease control in response to Brassicaceae SM soil amendment is attained through various biological and chemical functional pathways [6,12,60]. In this study, it was hypothesized that genes functional in pathogen defense and hormone signaling would be altered in response to SM application relative to the unamended control soil. Changes in the root transcriptome in response to these soil treatments were examined to assess the potential role of host genotype in modulating the disease control outcome. In addition, we hypothesized that alterations in host gene expression would be correlated with qualitative and/or quantitative changes in the rhizosphere-microbiome.

Upon pathogen infection, a number of integrated plant defense responses are typically activated to ward off the invader, including cell wall strengthening through synthesis of callose and lignin, pathogen cell wall degradation through synthesis of chitinases, glucanases, or pectinase, and accumulation of defense signaling components, such as pathogenesis related proteins [61,62,63,64]. Changes in plant hormone concentration and sensitivity can be triggered by biotic or abiotic stress conditions and subsequently mediate a wide range of adaptive responses [65]. Recent advances in characterization of plant immunity support the importance of phytohormones as primary signals in the regulation of plant defense responses [65,66]. In most cases, SA, JA, and ET interact antagonistically or synergistically as the backbone of the defense signaling network [67,68,69,70,71,72,73]. We find support for the hypothesis that phytohormone signaling and pathogen defense responses are not only treatment-dependent, but they are also largely genotype-dependent.

Expression patterns of genes in SM-amended soil and NTC soil were similar 48 h post-planting, regardless of rootstock genotype. However, by 72 h post-planting, there were distinctive transcriptional changes associated with cultivation in SM-amended soil and evidence of SM-mediated regulation of plant defense responses in both rootstock genotypes. For example, DEG involved in modulating phytohormone signaling pathways (e.g., auxin signaling and flavonoid biosynthesis in both genotypes; ET-mediated signaling in M.26; ABA-mediated signaling and suppression of JA signaling in G.210) and inducible defense responses (e.g., plant-pathogen interactions in both genotypes; chitin-induced immunity in M.26) had accumulated to higher levels in root tissue cultivated in SM-amended soil relative to the NTC. In contrast, defense responses in G.210 were elevated in NTC treatments relative to SM treatments at the 7-day timepoint. For example, log2 fold change values for defense-associated DEG expressed in the roots of plants grown in SM-amended soil generally ranged from 2–5 (Table 4 and Figure 2C). For G.210 cultivated in NTC soil, several DEG involved in phytohormone signaling and defense had log2 fold change values much greater than 5 by 72 h (e.g., MDP0000166337, MDP0000296675, and MDP0000729108) and 7 days (e.g., MDP0000193383, MDP0000313454, and MDP0000206106) (Table 4 and Figure 2C). The shift toward a stronger defense response at the 7-day timepoint in G.210 x NTC was also evident based upon a comparison of gene expression profiles of G.210 and M.26 rootstocks cultivated in NTC soil (Figure 6).

Plants are programmed to minimize expression of resistance mechanisms in the absence of a stress event [74]. Inducible defenses have evolved to save energy under enemy-free conditions and plants express these defenses if the benefits outweigh the costs of the resistance response [75]. Priming for defense may enhance the plants potential to successfully protect against pathogen invasion at minimal cost to plant production. In the absence of pathogen infection, the energy cost of chemical defense priming by exposure to low doses of β-aminobutyric acid only resulted in minor reductions in relative growth rate for Arabidopsis [75]. The relatively small amount of defense gene expression triggered by SM amendment may lead to more rapid activation (priming) of defense reactions if the plant host is subjected to pathogen challenge.

A small number of DEG [32] were altered in a similar manner at the same timepoint in both rootstock genotypes when cultivated in SM-amended soil. This finding indicated that SM soil amendment specifically induced small changes to the transcriptome relative to that of the same rootstock when planted in NTC soil. A study on hyperosmotic priming of Arabidopsis seedlings showed that a mild salt treatment resulted in only minor changes in histone levels but significantly altered the epigenome landscape [76], suggesting that environmental fluctuations may lead to small changes in gene expression but significant changes in phenotype. This unique subset of DEG detected for rootstocks cultivated in SM-amended soil contained upregulated genes involved in disease resistance, as well as genes which may be associated with a broad range of other activities taking place in the plant in response to this treatment (e.g., metal ion binding, response to stresses, and lipid transfer).

Our analysis provided evidence that ethylene-mediated transcription factors are likely to be important aspects of the multicomponent defense strategy in both rootstock genotypes when exposed to pathogen attack, irrespective of the soil treatment. Transcription factors (TF) play an essential role in gene regulation and link phytohormone signaling with downstream metabolic pathways generating antimicrobial compounds and PR proteins [20]. Although differing in time and the specific gene, DEG encoding putative ethylene response factors (ERF-RAP2-4) were elevated in both soil treatments, regardless of genotype (ERF-RAP2-4: M.26 x SM-72 h; ERF-RAP2-4: G.210 x SM-7 day; ERF 2: M.26 x NTC-72 h; ERF-RAP2-11: G.210 x NTC-72 h).

The question remains as to the means by which defense priming is triggered by SM, given that root infection by soil-borne pathogens was shown in this and previous studies to be suppressed by Brassicaceae SM [8,12,77,78,79]. In both rootstock genotypes, a higher number of DEG downregulated in SM treatment were involved in antioxidant defense and cell wall-related processes reflecting a reduction in pathogen infection and corresponding cellular damage in SM treatments. This outcome would be anticipated based upon reduced pathogen loads commonly realized in response to SM amendment, which was observed in this and previous studies [12,19]. Both genotypes also showed reduced transcription of MLO proteins at 72 h in SM (relative to NTC), a change which has been associated with reduced susceptibility to oomycete pathogens [80].

### 4.2. Transformation of the Rhizosphere Microbiome

Plant defense responses can be induced by several elements, including perception of pathogens [81], insect herbivores [82,83], chemical compounds [84,85,86], and plant beneficial microorganisms [87]. Therefore, an additional aim of this study was to explore whether alterations in host gene expression were temporally associated with changes in density and/or community composition of the rhizosphere-microbiome.

Transformation of the rhizosphere microbiome is critical for long-term pathogen suppression obtained in response to Brassicaceae SM amendment [8,12,78,88]. Specific changes in structure of the rhizosphere microbiome in response to SM function in control of various apple root pathogens with the microbial group(s) contributing to disease control often differing with the target pathogen [5,8]. It was previously proposed that amplification of *Streptomyces* apple rhizosphere populations in SM-amended soil contributed to the suppression of root infection by *R. solani* AG-5 via induction of host systemic resistance response [6,8]. Similarly, in the current study, relative abundance of *Streptomyces* was significantly amplified in the rhizosphere of SM-cultivated rootstocks in association with altered gene expression and disease control. The modified microbiome possessed several additional elements associated with suppression of soil-borne pathogens, nutrient cycling or degradation of recalcitrant chemical compounds. These included bacteria involved in nitrogen fixation, such as *Sinorhizobium* spp., pathogen suppression, such as *Lysobacter* spp. [89,90], and soil bioremediation, such as *Shinella* spp. [91] and *Arthrobacter* spp. [92].

After 7 days, M.26 cultivated in the NTC soil supported greater levels of fungi in the root zone than those cultivated in the SM-amended soil. Interestingly, SM was associated with a heightened level of fungal defense (Chitin catabolic process) in M.26 (but not in G.210) at 72 h. Thus, fungal pathogens may have more rapidly colonized susceptible M.26 roots in NTC soil and accumulated to higher levels in the root zone. The ARD susceptible rootstock M.26 grown in NTC soil has consistently been found to support higher fungal loads than the tolerant rootstock G.210 [9] and a greater relative abundance of fungi that function as root pathogens of apple, including *Ilyonectria robusta* [9,25,93]. In the current study, G.210 cultivated in SM-amended soil supported a greater density of fungi in root zones than in the NTC soil after 7 days. SM-associated rhizosphere fungal communities generally consist of a greater number of taxa with positive plant associations than the community in a corresponding untreated soil [9,12,19]. There is also evidence that Geneva rootstocks generally host higher levels of arbuscular mycorrhizal fungi (AMF), as compared to rootstocks in the Malling series [93]. AMF rely on carbon compounds derived from host plant photosynthesis and may play a role in displacing pathogenic fungi. As stated above, upregulated DEG in G.210 at 72 h mapped to KEGG pathways associated with starch and sucrose metabolism, Glycolysis/gluconeogenesis, and Galactose metabolism (Figure 3). It is also notable that 2 DEG associated with Carotenoid biosynthesis were upregulated at 72 h in G.210 (Table 4 and Figure 3). Carotenoid derived strigolactones have been implicated in presymbiotic signaling to AMF root colonization [94].

Unlike fungi, the quantity of bacterial DNA detected in the root zone did not appear to be treatment-dependent, suggesting that SM-mediated protection in the rhizosphere may have more to do with the changes in bacterial community composition noted above than density. There was, however, some evidence that transcriptional responses may have affected bacterial abundance in the rhizosphere of M.26. The large reduction in bacterial abundance which occurred in both NTC and SM treatments between 72 h and 7 days coincided with specific transcriptomic changes associated with the metabolism of sugars (M.26 x NTC), carbohydrates (M.26 x NTC), and amino acids (M.26 x NTC and SM), materials which are typically released in large quantities in root exudates (Figure 3A). Thus, the observed reduction in bacterial density at the 7-day timepoint both M.26 treatments may have been related to the reallocation of photosynthate toward defense-related activities (e.g., secondary metabolite production).

### 4.3. Genotype-Specific Transcriptome Response to Cultivation in NTC Replant Orchard Soil

Differential gene expression between the tolerant and susceptible rootstock in NTC soil revealed a number of defense-related changes occurring under pathogen pressure and provided insight into genotype-specific mechanisms of resistance. For example, genes linked to changes in cell wall integrity and the wounding response supported the hypothesis that M.26 was experiencing higher initial levels of cellular injury than G.210. At the same time, genes involved in cell wall organization and biogenesis were more highly expressed in G.210, a finding which may be related to the need to synthesize biomass components for rapidly dividing root cells.

Zhu et al. [24] demonstrated that, in the absence of pathogen challenge, the disease tolerant apple rootstock, G.935, possessed elevated transcript abundance for genes annotated to system-wide defense responses in root tissue relative to that detected in the disease susceptible rootstock B.9. When challenged with the root pathogen *Pythium ultimum*, transcript profiles for four specific defense related genes exhibited early (day 1) but transient induction in the susceptible rootstock [23]. In contrast, induction levels for these genes was higher in G.935 and persisted at later timepoints, including 7 and 14 days. Therefore, in addition to modulation of root architecture, we hypothesized that genotype-specific changes related to more rapid defense induction would be evident in G.210. In this case, when cultivated in NTC soil, we did not see clear evidence for more rapid defense induction by G.210 relative to M.26 at the 48- or 72-h timepoints. Biological processes related to defense (including increased expression of genes involved in hypersensitive responses) were not elevated in G.210 relative to M.26 until the 7-day timepoint, which is similar to the previous findings of Zhu et al. [23,24]. Likewise, in a transcriptome study on grapevine resistance to powdery mildew, the authors found evidence for strong induction of the basal defense response in the susceptible genotype, while the transcriptome of the resistant genotype responded weakly to powdery mildew infection [95]. In contrast, there was little evidence that G.210 was redirecting resources towards basal defense mechanisms at this later timepoint in SM-amended soil. Along these same lines, by 7 days, NTC and SM-amended transcriptomes had largely diverged in G.210, and there was a large accumulation of genes related to defense which were expressed at higher levels in the NTC relative to SM-amended soil (i.e., downregulated). However, in M.26, NTC and SM-amended transcriptomes were largely identical at 7 days. This was surprising considering that by 72 h, a number of DEG involved in phytohormone signaling and/or the utilization of secondary metabolites had accumulated to higher levels in M.26 root tissue cultivated in NTC, in comparison to SM-amended soil. However, the transient nature of the response in the susceptible rootstock is in concordance with findings that were observed when the susceptible B.9 rootstock was challenged with *P. ultimum* [23]. 

## 5. Conclusions

This study demonstrates the significant effect of an organic plant residue soil amendment on the apple root transcriptome and on rhizosphere microbial community dynamics. The results of this study support the hypothesis that both apple rootstock genotypes benefitted from the SM soil amendment in multiple manners, including: SM-mediated defense responses, prophylactic alterations to the root-associated microbiome, and reduced pathogen loads in comparison to the NTC. More specifically, these findings suggest that SM-dependent transcriptional changes may protect plants by priming them for improved performance during subsequent (and more intensive) pathogen-induced cellular defense responses. However, further studies are necessary to improve our understanding of exactly how SM influences plant defense mechanisms in different apple rootstock genotypes. The patterns revealed by differential gene expression analysis also provided new insight into how ARD “susceptible” versus “tolerant” rootstock cultivars may differ in their response to pathogen pressure. Genotype-specific trade-offs in energy allocation largely influenced the timing of induced defense responses in NTC soil. We propose that, upon exposure to ARD-pathogens, G.210 initially allocates more energy to the production of new root biomass, while M.26 directs resources towards defense and secondary metabolite production. Overall, findings from this study will be of value for rootstock breeding through identification of genes that may confer host tolerance/resistance and optimization of implementation strategies for alternative soil-borne disease management programs that do not utilize pre-plant soil fumigation.

## Figures and Tables

**Table 1:** Comparison groups and the number of identified differentially expressed genes (DEG).

Sampling Timepoint	Apple Rootstock Genotype	Comparisons ^z^	No. of DEG	No. of Upregulated ^y^	No. of Downregulated
48 h	M.26	M.26-48 h-NTC vs. M.26 48 h-SM	7	6	1
	G.210	G.210-48 h-NTC vs. G.210-48 h-SM	6	2	4
72 h	M.26	M.26-72 h-NTC vs. M.26 72 h-SM	122	64	58
	G.210	G.210-72 h-NTC vs. G.210-72 h-SM	177	106	71
7 d	M.26	M.26-7 d-NTC vs. M.26-7 d-SM	10	6	4
	G.210	G.210-7 d-NTC vs. G.210-7 d-SM	232	33	199

^z^ NTC = no treatment control; SM = Brassicaceae seed meal formulation of Brassica juncea/Sinapis alba (1:1) at a rate of 4.4 t ha^−1^, ^y^ DEG were categorized as upregulated where expression was higher in plants cultivated in seed meal (SM)-amended soil relative to that observed in plants cultivated in the no treatment control soil. Downregulated indicates that expression was higher in plants cultivated in control versus SM-amended soil.

**Table 2:** Selected genes differentially expressed in apple rootstock M.26 when planted in SM-amended replant orchard soil relative to non-treated orchard soil, with annotated function.

Gene ID in GDR ^z^	Protein Function Annotation ^y^	Log_2_(FC) ^x^	Timepoint
MDP0000196909	TMV resistance protein N	2.03	48 h
MDP0000711750	CBL-interacting serine/threonine-protein kinase 12	−2.61	48 h
MDP0000306089	Calcium-binding EF-hand family protein	2.33	72 h
MDP0000283789	Disease resistance protein RGA3	1.94	72 h
MDP0000162375	Disease resistance protein	2.31	72 h
MDP0000683814	Ethylene-responsive transcription factor RAP2-4	2.49	72 h
MDP0000278380	Pathogenesis-related protein 5	2.88	72 h
MDP0000198976	Serine/threonine-protein kinase CTR1	5.56	72 h
MDP0000273596	Serine/threonine-protein kinase PBL27	2.17	72 h
MDP0000268026	TMV resistance protein N	3.33	72 h
MDP0000191786	4-coumarate-CoA ligase-like 5	−5.46	72 h
MDP0000185169	Benzyl alcohol O-benzoyltransferase	−5.45	72 h
MDP0000189033	Ethylene-responsive transcription factor 2	−4.13	72 h
MDP0000234530	MLO-like protein 6	−10.06	72 h
MDP0000266840	NADP-dependent alkenal double bond reductase P2	−2.66	72 h
MDP0000231619	Probable pectinesterase/pectinesterase inhibitor 7	−5.13	72 h
MDP0000318604	Probable S-adenosylmethionine-dependent methyltransferase	−2.85	72 h
MDP0000675952	Probable S-adenosylmethionine-dependent methyltransferase	−4.2	72 h
MDP0000931970	Receptor-like protein kinase THESEUS 1	−2.38	72 h
MDP0000755567	Indole-3-acetic acid-induced protein ARG2	2.29	7 d

^z^ Apple reference genome “Malus_x_domestica.v1.0.contigs.gff” was obtained from rosaceae.org, accessed on 24 February 2021, ^y^ Protein function annotation is based on the blastX search against UniprotKB/Swiss-Prot (swissprot) database from NCBI, ^x^ Variations in transcript abundance were based on comparison between control and seed meal treatments, and expressed as log_2_ fold change; (+) indicates upregulated, and (−) indicates downregulated, in rootstocks cultivated in seed meal-treated soil.

**Table 3:** Selected genes differentially expressed in apple rootstock G.210 when planted in SM-amended replant orchard soil relative to non-treated orchard soil, with annotated function.

Gene ID in GDR ^z^	Protein Function Annotation ^y^	Log_2_(FC) ^x^	Timepoint
MDP0000319359	Calcium-dependent lipid-binding (CaLB domain) family protein	6.41	48 h
MDP0000296303	Abscisic acid-insensitive 5-like protein	2.17	72 h
MDP0000306089	Calcium-binding EF-hand family protein	2.51	72 h
MDP0000216765	CBL-interacting serine/threonine-protein kinase 9	2.19	72 h
MDP0000127009	Disease resistance protein RGA2	1.82	72 h
MDP0000137225	Disease resistance protein	3.02	72 h
MDP0000640906	Disease resistance protein	2.95	72 h
MDP0000162375	Disease resistance protein	2.22	72 h
MDP0000228070	9-cis-epoxycarotenoid dioxygenase	8.60	72 h
MDP0000929213	9-cis-epoxycarotenoid dioxygenase	3.68	72 h
MDP0000484601	GTP-binding protein brassinazole insensitive pale green 2	1.89	72 h
MDP0000290028	LRR receptor-like serine/threonine-protein kinase GSO2	4.90	72 h
MDP0000119875	Ninja-family protein AFP2	2.37	72 h
MDP0000875341	Ninja-family protein AFP3	4.79	72 h
MDP0000588503	Pathogenesis-related protein 10	2.18	72 h
MDP0000234689	S-type anion channel SLAH3	3.23	72 h
MDP0000268026	TMV resistance protein N	2.59	72 h
MDP0000657441	WRKY transcription factor 68	2.47	72 h
MDP0000166337	Abscisic acid 8′-hydroxylase 4	−9.91	72 h
MDP0000197409	Carotenoid cleavage dioxygenase 7	−3.39	72 h
MDP0000139334	Carotenoid cleavage dioxygenase 7	−3.47	72 h
MDP0000652331	Cytochrome b561 and DOMON	−2.24	72 h
MDP0000138669	Ethylene-responsive transcription factor RAP2-11	−6.41	72 h
MDP0000295857	Fusaric acid resistance protein	−4.77	72 h
MDP0000239643	MLO-like protein 6	−3.88	72 h
MDP0000266930	NADPH-dependent oxidoreductase 2-alkenal reductase	−3.17	72 h
MDP0000872370	NADPH-dependent oxidoreductase 2-alkenal reductase	−3.00	72 h
MDP0000675952	Probable S-adenosylmethionine-dependent methyltransferase	−5.92	72 h
MDP0000261194	Probable S-adenosylmethionine-dependent methyltransferase	−6.22	72 h
MDP0000729108	Pathogenesis-related protein 10	−11.18	72 h
MDP0000296675	Protein LURP-one-related 4	−∞	72 h
MDP0000930978	Protein SAR deficient 1	−10.33	72 h
MDP0000139821	Disease resistance protein	−2.48/−2.32	72 h/7 d
MDP0000309171	Disease resistance protein	−2.82/−3.34	72 h/7 d
MDP0000792101	Wound-induced protein	−5.45/−5.61	72 h/7 d
MDP0000275716	Cellulose synthase-like protein B4	−3.35	7 d
MDP0000411073	Cellulose synthase-like protein E2	−3.91	7 d
MDP0000221346	Cellulose synthase-like protein G2	−3.33	7 d
MDP0000314000	Cysteine-rich receptor-like protein kinase 29	−3.43	7 d
MDP0000685403	Cysteine-rich receptor-like protein kinase 29	−10.06	7 d
MDP0000927688	Cysteine-rich repeat secretory protein 38	−7.61	7 d
MDP0000416561	Cysteine-rich repeat secretory protein 38	−8.13	7 d
MDP0000842997	ABC transporter C family member 3	−2.79	7 d
MDP0000865714	Disease resistance protein	−16.43	7 d
MDP0000706371	Disease resistance protein	−2.73	7 d
MDP0000126026	Disease resistance protein	−3.37	7 d
MDP0000224187	Disease resistance RPP13-like protein 4	−3.27	7 d
MDP0000193383	Endochitinase EP3	−10.32	7 d
MDP0000143462	Glycine-rich cell wall structural protein	−13.37	7 d
MDP0000260110	Major allergen Mal d 1	−3.06	7 d
MDP0000831518	Major allergen Mal d 1	−4.14	7 d
MDP0000831519	Major allergen Mal d 1	−4.46	7 d
MDP0000295542	Major allergen Mal d 1	−5.61	7 d
MDP0000864747	Major allergen Mal d 1	−7.23	7 d
MDP0000313454	Major allergen Pru ar 1	−9.78	7 d
MDP0000124524	Pathogenesis-related protein STH-2	−6.88	7 d
MDP0000119517	Pathogenesis-related protein STH-2	−7.37	7 d
MDP0000221319	Pectin acetylesterase 8	−7.66	7 d
MDP0000249386	Polygalacturonase	3.07	7 d
MDP0000755567	Indole-3-acetic acid-induced protein ARG2	2.39	7 d
MDP0000683814	Ethylene-responsive transcription factor RAP2-4	2.17	7 d
MDP0000282421	U-box domain-containing protein 9	1.94	7 d
MDP0000897962	Probable LRR receptor-like serine/threonine-protein kinase	−2.68	7 d
MDP0000229843	Protein DMR6-like oxygenase	−5.85	7 d
MDP0000576922	Protein DMR6-like oxygenase	−3.28	7 d
MDP0000218810	Protein DMR6-like oxygenase	−3.34	7 d
MDP0000593536	Protein DMR6-like oxygenase	−3.67	7 d
MDP0000566057	Protein DMR6-like oxygenase	−4.53	7 d
MDP0000147913	Protein DMR6-like oxygenase	−5.70	7 d
MDP0000694318	Rust resistance kinase Lr10	−5.93	7 d
MDP0000156351	U-box domain-containing protein 21	−4.99	7 d
MDP0000206106	Wall-associated receptor kinase	−7.21	7 d

^z^ Apple reference genome “Malus_x_domestica.v1.0.contigs.gff” was obtained from rosaceae.org, accessed on 24 February 2021, ^y^ Protein function annotation is based on the blastX search against UniprotKB/Swiss-Prot (swissprot) database from NCBI, ^x^ Variations in transcript abundance were based on comparison between control and seed meal treatments, and expressed as log_2_ fold change; (+) indicates upregulated, and (−) indicates downregulated, in rootstocks cultivated in seed meal-treated soil.

**Table 4:** Functional annotation of genes exhibiting comparable pattern of differential expression between seed meal and non-treated replant orchard soils for both apple rootstock genotypes (M.26 and G.210).

Gene ID in GDR ^z^	Log_2_(FC) ^y^ in		Biological Process ^x^	Protein Function Annotation ^w^
	M.26	G.210		
MDP0000306089	2.33	2.51	Plant-pathogen interaction	Calcium-binding EF-hand family protein
MDP0000162375	2.31	2.22	Disease resistance	Disease resistance protein
MDP0000268026	3.33	2.59	Protein binding	TMV resistance protein N
MDP0000302115	4.12	3.92	N/A ^v^	Disease resistance protein
MDP0000570102	2.09	2.51	Flavonoid biosynthetic process	N/A
MDP0000755567	2.29	2.39	Response to stress	Late embryogenesis abundant like-5 (ATLEA5)
MDP0000385497	2.14	2.12	Response to stress	Late embryogenesis abundant like-5 (ATLEA5)
MDP0000234689	2.62	3.23	Response to salt stress and water deprivation	SLAC1 homologue 3 (SLAH3)
MDP0000190029	2.06	2.06	DNA binding	Nuclear factor Y, Subunit C2
MDP0000581293	2.76	2.12	DNA binding	REVEILLE 1-like
MDP0000153123	1.97	2.40	Metal ion binding	Heme-binding protein
MDP0000821892	1.91	2.34	Metal ion binding	Metallothionein
MDP0000212510	2.83	2.23	Sugar transport	Monosaccharid transporter 2
MDP0000281884	2.70	2.56	Sugar transport	Monosaccharid transporter 2
MDP0000403033	2.38	2.61	Ion channel	Chloride channel C
MDP0000307717	3.06	3.60	Lipid transfer	Lipid-transfer protein
MDP0000270246	2.45	2.13	Protein insertion into membrane	Membrane protein
MDP0000322201	2.69	2.44	N-acetyltransferase	Acyl-CoA N-acyltransferases (NAT) superfamily protein
MDP0000210084	2.77	2.94	Involved in leaf vasculature patterning	Glycine-rich protein DOT1-like
MDP0000827125	2.59	3.60	N/A	N/A
MDP0000286637	2.62	3.24	Transport	Integral membrane HPP family protein
MDP0000239643	−3.99	−3.88	Cell death	MLO family protein
MDP0000171573	−3.09	−2.63	Fructose and mannose metabolism	GroES-like zinc-binding alcohol dehydrogenase family protein
MDP0000145305	−2.81	−3.75	DNA replication initiation	Dienelactone hydrolase
MDP0000675952	−4.20	−5.92	Methyltransfer	Methyltransferase
MDP0000258061	−6.21	−6.46	Oxidation-reduction process	2-oxoglutarate (2OG) and Fe(II)-dependent oxygenase superfamily protein
MDP0000139947	−6.11	−5.058	Protein phosphorylation	Protein kinase THE1
MDP0000870778	−2.14	−2.02	Protein phosphorylation	Receptor-like protein kinase FERONIA
MDP0000252274	-∞	−2.61	Isoprenoid biosynthetic process	Terpenoid synthase
MDP0000295857	−5.02	−4.77	Phosphate ion transmembrane transport	P-hydroxybenzoic acid efflux pump subunit
MDP0000464148	−3.01	−3.18	Integral component of membrane	Transmembrane protein
MDP0000816071	−7.76	−6.15	N/A	LOB domain-containing protein

^z^ Apple reference genome “Malus_x_domestica.v1.0.contigs.gff” was obtained from rosaceae.org, accessed on 24 February 2021, ^y^ Variations in transcript abundance were based on comparison between control and seed meal treatments, and expressed as log_2_ fold change; (+) indicates upregulated, and (−) indicates downregulated, in the root of rootstocks cultivated in seed meal-treated soil, ^x^ Gene ontology biological process annotation was based on Malus_x_domestica_v1.0.genes2GO from rosaceae.org, accessed on 24 February 2021. ^w^ Protein function annotation is based on the blastX search against UniprotKB/Swiss-Prot (swissprot) database from NCBI. ^v^ N/A = no annotated function.

## Data Availability

The datasets generated and analyzed during the current study are available in the Figshare repository (10.6084/m9.figshare.14219483) and include unedited 16 S rRNA OTU read count tables, as well as non-normalized raw counts of the sequencing reads, which were used for the differential expression analysis. Any other data generated or analyzed during this study are included in this published article and its supplementary information files or are available from the corresponding author upon reasonable request.

## Supplementary Materials

The following are available online at https://www.mdpi.com/article/10.3390/microorganisms9040763/s1, Figure S1: Validation of differential expression patterns for selected differentially expressed genes in apple rootstock G.210 by qRT-PCR, Figure S2: Validation of differential expression patterns for selected differentially expressed genes in apple rootstock M.26 by qRT-PCR, Table S1: Statistics of RNA-seq read mapping, Table S2: Significant GO Biological Process terms (*p* < 0.05) over-represented in M.26 relative to G.210, Table S3: Significant GO Biological Process terms (*p* < 0.05) over-represented in G.210 relative to M.26.
