# Supplementary material for: Comparative Analysis of the Apple Root Transcriptome as Affected by Rootstock Genotype and Brassicaceae Seed Meal Soil Amendment: Implications for Plant Health
Source: Microorganisms. 2021 Apr 6;9(4):763. doi: 10.3390/microorganisms9040763 (PMC8067487; doi:10.3390/microorganisms9040763)
Supplement: Supplementary file 1 [file microorganisms-09-00763-s001.zip › Wang et al. Supplementary/Suppl Table1.docx]

**Table S1.** Statistics of RNA-seq read mapping^z^

| Overall alignment (percentage) |  |  | No. of reads | Percentage |
| --- | --- | --- | --- | --- |
| Mapped reads (92%) | Exon (69%) | Uniquely mapped reads | 576,614,872 | 77 |
|  |  | Non-specifically mapped reads | 174,974,915 | 23 |
|  |  |  |  |  |
|  | Intron (0%) | Uniquely mapped reads | 875 | 92 |
|  |  | Non-specifically mapped reads | 78 | 8 |
|  |  |  |  |  |
|  | Intergenic (31%) | Uniquely mapped reads | 261,938,151 | 79 |
|  |  | Non-specifically mapped reads | 74,232,400 | 21 |
|  |  |  |  |  |
| Un-mapped reads (8%) |  |  | 94,587,938 | 8 |
| Total reads |  |  | 1,182,349,229 |  |

^z^Apple reference genome Malus_x_domestica.v1.0.contigs.gff” was obtained from rosaceae.org.
