# Supplementary material for: Comparative Analysis of the Apple Root Transcriptome as Affected by Rootstock Genotype and Brassicaceae Seed Meal Soil Amendment: Implications for Plant Health
Source: Microorganisms. 2021 Apr 6;9(4):763. doi: 10.3390/microorganisms9040763 (PMC8067487; doi:10.3390/microorganisms9040763)
Supplement: Supplementary file 1 [file microorganisms-09-00763-s001.zip › Wang et al. Supplementary/Supplementary figures.docx]

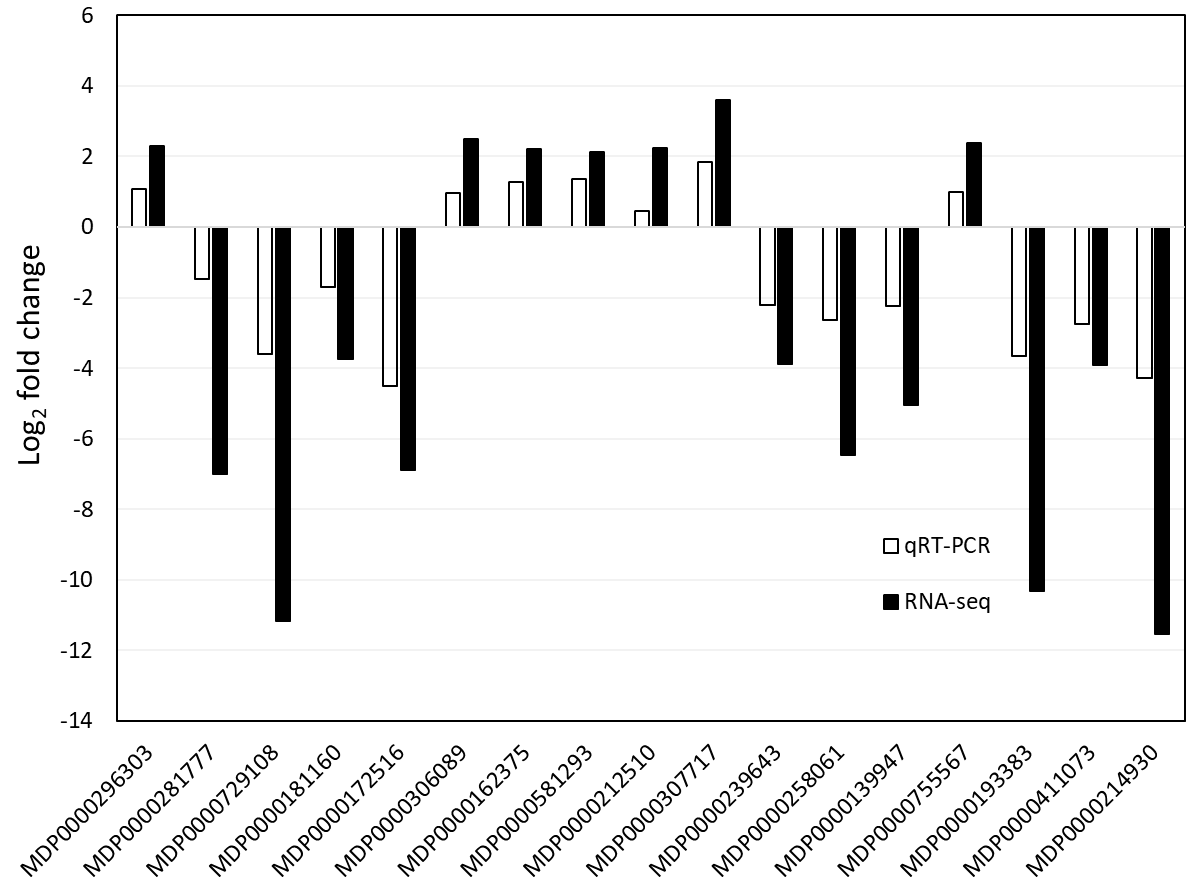


**Figure S1.** Validation of differential expression patterns for selected differentially expressed genes in apple rootstock G.210 by qRT-PCR. Gene expression was shown in y-axis. The values of log_2_ fold change from RNA-seq data (grey) were validated through comparing qRT-PCR amplification value of control (black) and seed meal (white). The upward and downward directions for RNA-seq data represent upregulated and downregulated gene expression in seed meal treatment relative to the non-treated control, respectively. Seed meal = Brassica juncea/Sinapis alba seed meal formulation (1:1); Control = no treatment control.

*
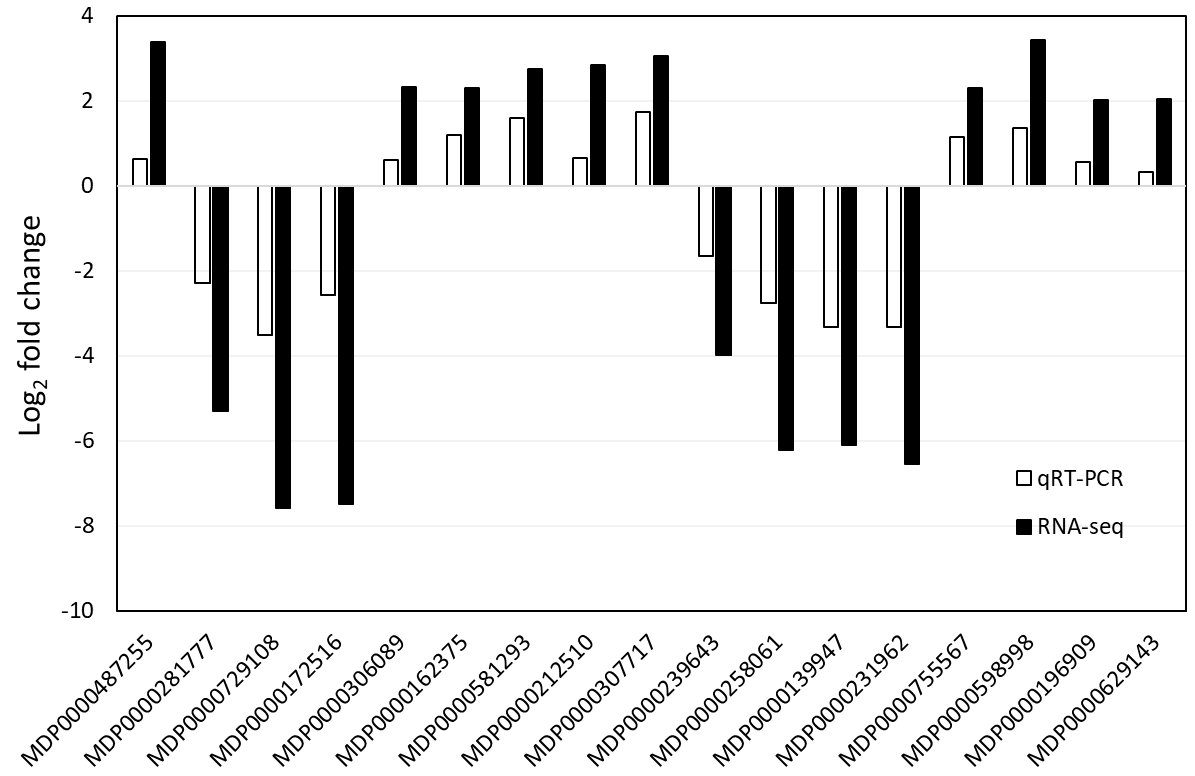
*

**Figure S2.** Validation of differential expression patterns for selected differentially expressed genes in apple rootstock M.26 by qRT-PCR. Gene expression was shown in y-axis. The values of log_2_ fold change from RNA-seq data (grey) were validated through comparing qRT-PCR amplification value of control (black) and seed meal (white). The upward and downward directions for RNA-seq data represent upregulated and downregulated gene expression in seed meal treatment relative to the non-treated control, respectively. Seed meal = Brassica juncea/Sinapis alba seed meal formulation (1:1); Control = no treatment control.
